# ERK Hyperactivation Serves as a Unified Mechanism of Escape in Intrinsic and Acquired CDK4/6 Inhibitor Resistance in Acral Lentiginous Melanoma

**DOI:** 10.21203/rs.3.rs-2817876/v1

**Published:** 2023-04-20

**Authors:** Vito Rebecca, Kasturee Jagirdar, Marie Portuallo, Meihan Wei, Matthew Wilhide, Jeremy Bravo, Bailey Robertson, Gretchen Alicea, Crsytal Aguh, Min Xiao, Tetiana Godok, Dylan Fingerman, Gregory Brown, Meenhard Herlyn, Brian Guo, Eneda Toska, Daniel Zabransky, Bradley Wubbenhorst, Katherine Nathanson, Shawn Kwatra, Yogesh Goyal, Hongkai Ji, Qin Liu

**Affiliations:** Johns Hopkins University; The Wistar Institute; Johns Hopkins; Johns Hopkins University; University of Pennsylvania; The Wistar Institute

## Abstract

Patients with metastatic acral lentiginous melanoma (ALM) suffer worse outcomes relative to patients with other forms of cutaneous melanoma (CM), and do not benefit as well to approved melanoma therapies. Identification of cyclin-dependent kinase 4 and 6 (CDK4/6) pathway gene alterations in > 60% of ALMs has led to clinical trials of the CDK4/6 inhibitor (CDK4i/6i) palbociclib for ALM; however, median progression free survival with CDK4i/6i treatment was only 2.2 months, suggesting existence of resistance mechanisms. Therapy resistance in ALM remains poorly understood; here we report hyperactivation of MAPK signaling and elevated cyclin D1 expression are a unified mechanism of both intrinsic and acquired CDK4i/6i resistance. MEK and/or ERK inhibition increases CDK4i/6i efficacy in a patient-derived xenograft (PDX) model of ALM and promotes a defective DNA repair, cell cycle arrested and apoptotic program. Notably, gene alterations poorly correlate with protein expression of cell cycle proteins in ALM or efficacy of CDK4i/6i, urging additional strategies when stratifying patients for CDK4i/6i trial inclusion. Concurrent targeting of the MAPK pathway and CDK4/6 represents a new approach to improve outcomes for patients with advanced ALM.

## Introduction

Acral lentiginous melanoma (ALM) constitutes a distinct disease relative to other forms of cutaneous melanoma (CM) (i.e., superficial spreading, nodular, lentigo maligna) due, in part, to a different cell of origin (volar versus non-volar skin melanocytes) (1), the defining acral skin sites they arise on, and a complex genomic landscape (2). Although patients with metastatic ALM suffer worse outcomes relative to patients with other subtypes of CM, the underlying molecular mechanisms responsible for ALM initiation, progression, and therapy resistance remain poorly understood (3, 4). Existing standard-of-care targeted therapies (i.e., BRAF inhibitors) for CM are not available to the majority of advanced ALM patients due to a lower frequency of *BRAF*^V600E/K^ mutations (20% versus 50% in other forms of CM (2)). Further, targeted therapy is not as effective in ALM patients with *BRAF*^V600E/K^ mutations (5). Additionally, the efficacy of immune checkpoint blockade (ICB) is less effective and remains poorly understood in ALM (2, 5, 6). Therefore, new therapy strategies tailored to the ALM patient population are critically warranted.

Recent genetic characterization of ALM patient tumor tissue has identified cyclin-dependent kinase 4 (CDK4)-pathway (e.g., *CDK4* amplification, *CDK6* amplification, *CCND1* amplification, *P16*^INK4A^ loss) alterations in 53–82% of ALM cases (2, 7), with *CDK4* amplification and *P16*^INK4A^ loss each independently serving as predictors of shorter patient overall survival. CDKs propel cell cycle progression and their frequent dysregulation in cancer contributes to the uncontrolled cellular proliferation, which is regarded as one of the hallmarks of cancer. ALM cell lines and patient-derived xenograft (PDX) models with CDK4 pathway alterations were reported to exhibit elevated *in vivo* sensitivity to CDK4/6 inhibitors (CDK4i/6i) (7), which provided rationale for the first phase II clinical trial (NCT03454919) of palbociclib in patients with advanced ALM whose tumors exhibit CDK4-pathway aberrations (8). Unfortunately, most patients did not benefit from palbociclib monotherapy, and the median progression free survival (mPFS) was only 2.2 months. The few patients who did respond did not often experience durable tumor control, suggesting that both intrinsic- and acquired-resistance mechanisms to single-agent palbociclib arise rapidly. To date, mechanisms of resistance to CDK4i/6i in ALM remain poorly understood.

Here, we identify hyperactivation of the mitogen-activated protein kinase (MAPK) pathway and elevated cyclin D1 as functional drivers of intrinsic and acquired CDK4i/6i resistance. The MAPK pathway sustains cyclin D1 levels, and we find in the context of intrinsic and acquired resistance to CDK4i/6i, elevated MAPK activity promotes ALM addiction to cyclin D1, which can be overcome with use of the clinical MEK inhibitor trametinib or genetic silencing of cyclin D1. Altogether, these findings represent the seminal report of a unified intrinsic and acquired CDK4i/6i resistance mechanism in ALM and conclude the addition of a MEK inhibitor may increase the durability of first- and second-line CDK4i/6i therapy in patients with advanced ALM.

## Results

### Genetic status of CDK4-pathway nodes does not predict protein expression or CDK4i/6i durability in ALM.

The current strategy for clinical use of CDK4i/6i in patients with advanced ALM rests upon the genetic status of CDK pathway nodes, in part due to recent evidence that *Cdk4* and/or *P16*^*INK4a*^ copy number status may be of prognostic significance for ALM patients (7); stemming from this study, only patients with *CDK4* gain, *CCND1* gain and/or *CDKN2A* loss were eligible for treatment with palbociclib (8). In an independent analysis of a separate ALM patient cohort, we find no significant correlation between the overall survival of ALM patients based off of *Cdk4* gain or *P16*^*INK4a*^ loss than those without *Cdk4* gain or *P16*^*INK4a*^, respectively (**Supplemental Fig. 1A, 1B** (9)). These data suggest the prognostic significance of *CDK4* pathway nodes may differ across patient cohorts.

**Figure 1:**
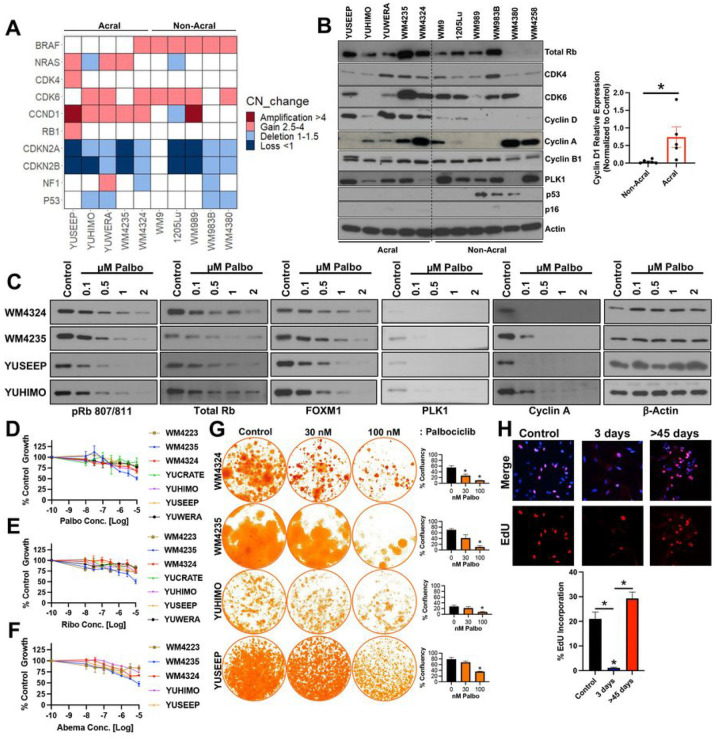
Genetic status of CDK4-pathway nodes does not predict protein expression or durability of CDK4/6 inhibition. (**A**) Copy number variation and mutational status was assessed across a panel of ALM and non-ALM models. (**B**) Western blot showing basal expression of cell cycle proteins across a panel of ALM and non-ALM models. Shown in the right panel is a densitometric quantification of cyclin D1 expression. (**C**) Cells were treated with increasing concentrations of palbociclib for 24 hrs before Western blotting. (**D**) A panel of ALM models were treated with increasing concentrations of palbociclib, (**E**) ribociclib, or (**F**) abemaciclib for 72 hrs before cell numbers were quantified using MTT. Bars show S.E. mean. (**G**) A panel of ALM cell lines were treated with palbociclib for 3–4 weeks before colonies were fixed and stained with crystal violet. Photographs are representative of three independent experiments and relative clonogenic survival quantitation is shown to the right. (**H**) WM4324 cells were treated with palbociclib (500 nM) for the time shown before EdU incorporation and imaging to assess cell proliferation. *p<0.05 and n^3^3 unless otherwise stated throughout panels.

We next characterized the relationship between copy number variations, mRNA levels, and baseline protein expression of CDK4 pathway nodes across a genetically diverse panel of human ALM and non-ALM cell lines (Fig. 1A, 1B). In agreement with clinical observations, CDK4 pathway nodes (*CDK4*, *CDK6*, *CCND1*, *CDKN2A*, *CDKN2B*) were highly dysregulated across our ALM models with evidence of CCND1, a key activator of CDK4 and CDK6, also being elevated at the protein level in ALM versus non-ALM models (Fig. 1B). Notably, there was no consistent agreement between the genomic information and protein expression of CDK4, CDK6, or cyclin D1 in our ALM panel. ALM cell lines with *CDK4*, *CDK6*, or *CCND1* genomic and transcriptomic amplifications did not robustly display elevated CDK4, CDK6, or cyclin D1 protein expression relative to ALM models with normal gene copy numbers, respectively (**Supplemental Fig. 1C, 1D, 1E**). Analysis of the TCGA to understand the relationship between copy number status, mRNA level, and protein expression for cyclin D1 in patients with superficial spreading melanoma (CDK4 and CDK6 protein expression unavailable) also revealed no correlation between genomic or transcriptomic status with protein expression (**Supplemental Fig. 1F**). This may serve as a cautionary note for the identification of ALM patients who may benefit from CDK4i/6i based solely on tumor sequencing, which may prevent patients with elevated CDK4/CDK6 protein expression but no clear evidence of copy number variation in CDK4/6 pathway genes from treatment.

In agreement with the literature, treatment with the CDK4i/6i palbociclib, ribociclib, or abemaciclib potently inhibits CDK4/6 substrates (p-Rb, FOXM1), and E2F target proteins (PLK1, cyclin A) in a dose-dependent manner (Fig. 1C, **Supplemental Fig. 1G, 1H**). Despite potent CDK4/6 and E2F suppression, CDK4i/6i treatment elicited a predominately cytostatic effect, with a subpopulation of viable ALM cells remaining after short-term treatment over the course of three days in the presence of non-physiologically high concentrations of three clinically utilized CDK4i/6i’s palbociclib (> 230 nM (10)) (Fig. 1D), ribociclib (> 1 mM (11)) (Fig. 1E) and abemaciclib (> 290 nM (12)) (Fig. 1F). When the treatment period is extended, therapy-resistant colonies continue to survive following > 3-weeks of chronic exposure to CDK4i/6i (Fig. 1G). Accordingly, CDK4i/6i elicited an initial inhibition of proliferative capacity followed by reignition of cell cycle progression following long-term treatment as seen by EdU incorporation (Fig. 1H).

CDK6 protein expression has been recently demonstrated to indirectly predict for sensitivity to CDK4/6 inhibition in ER^+^ breast cancers, non-small cell lung carcinomas, colorectal carcinomas, and superficial spreading melanomas (13). In contrast, an analysis of correlations for ALM sensitivity to CDK4i/6i revealed that CDK6 expression trends (p = 0.087) directly with palbociclib sensitivity in ALM (**Supplemental Fig. 1I**). The expression of CDK4 (p = 0.72) and Cyclin D1 (p = 0.6) did not correlate with ALM sensitivity to CDK4i/6i (**Supplemental Fig. 1J, 1K**). Alongside the first phase II clinical trial results of CDK4i/6i treatment in patients with advanced ALM, it was proposed that low MCM7 expression and *SH2B3* amplification serve as predictive biomarkers of poor response to CDK4i/6i (8). We find across our ALM cell line panel that sensitivity to CDK4i/6i with abemaciclib trended, albeit not significantly (p = 0.057), with baseline expression of MCM7 (**Supplemental Fig. 2A,2B**). In contrast, we did not observe significant relationships between palbociclib and ribociclib sensitivity with MCM7 expression, nor did we observe a correlation between any of the 3 clinically available CDK4i/6i tested with SH2B3 expression (**Supplemental Fig. 2B,2C**). We next put into context our CDK4i/6i data with recent reports of potential oncogenic drivers of ALM. LZTR1, an adaptor for Cullin 3 ubiquitin ligase complexes, was proposed to serve as a driver of ALM aggressiveness (9). No significant relationship emerged between LZTR1 expression and CDK4i/6i sensitivity (**Supplemental Fig. 2D**). Further, CRKL, a signaling adaptor protein in pathways including the IGF1R-PI3K axis, was also recently proposed to serve as an oncogenic driver of ALM (14). We also did not observe a significant correlation between *CRKL* expression and CDK4i/6i sensitivity (**Supplemental Fig. 2E**). Altogether, these data demonstrate that the protein expression of CDK4, CDK6, and cyclin D1 do not correlate with the respective gene copy number status, and the sensitivity of ALM cells to single-agent CDK4i/6i does not correlate to: a) *CDK4*, *CDK6*, or *CCND1* amplification or protein expression, b) reported CDK4i/6i sensitivity biomarkers (MCM7, SH2B3), or c) proposed ALM oncogenic drivers (CRKL, LZTR1).

### Loss of DUSP4 expression following CDK4i/6i promotes ERK activation and drives intrinsic resistance via cyclin D1.

**Figure 2:**
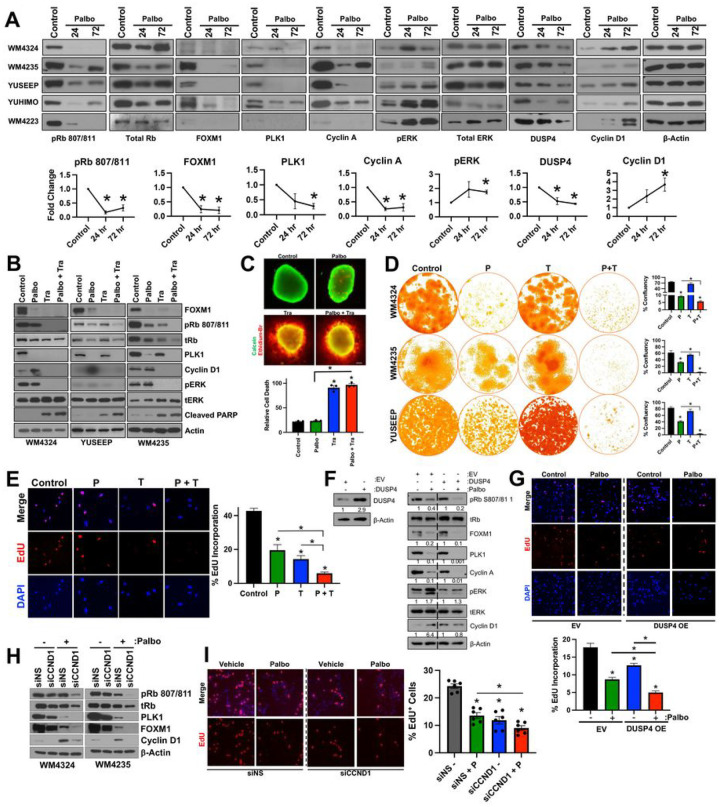
ERK hyperactivation drives intrinsic CDK4i/6i resistance via cyclin D1. (**A**) A panel of ALM cell lines were treated with palbociclib (500 nM, 24–72 hrs) before characterization by Western blotting (top panel). Densitometric analysis is shown in the bottom panel. (**B**) Cells were treated with palbociclib (500 nM) and/or trametinib (10 nM for WM4324/WM4235, 1 nM for YUSEEP) for 72 hrs before characterization by Western blotting. (**C**) WM4324 spheroids were formed before implantation in collagen and treatment with palbociclib and/or trametinib for 72 hours. Spheroids were subsequently stained with a viability stain and imaged by fluorescent microscopy (green indicates living cells, red indicates dead cells). (**D**) Cells were treated for up to 4 weeks with palbociclib (30 nM) and/or trametinib (0.3 nM) before colonies were fixed and stained with crystal violet. Quantification is shown in the right panel. (**E**) WM4235 cells were treated with palbociclib (500 nM) and/or trametinib (10 nM) for 24 hrs before subsequent staining with EdU. Shown in the panel to the right is quantification. (**F**) YUHIMO cells were stably transfected with either Empty Vector (EV) or DUSP4 (DUSP4 OE) in the presence or absence of palbociclib (500 nM) for 72 hrs before characterization by Western blotting. (**G**) YUHIMO cells were transfected with either EV or DUSP4 for 72 hrs before treatment with palbociclib (500 nM) for 72 hrs before proliferative capacity following EdU staining. (**H**) Cells were transfected with either non-specific siRNA (siNS) or siCCND1 in the presence or absence of palbociclib (500 nM) for 72 hrs before characterization by Western blotting. (**I**) WM4324 cells were transfected with either non-specific siNS or siCCND1 for 48 hrs before treatment with palbociclib (500 nM) for 6 hrs before proliferative capacity following EdU staining. *p<0.05 and n^3^3 unless otherwise stated throughout panels.

Improvement in the efficacy of CDK4i/6i with inhibitors of the MAPK pathway has been reported in prostate adenocarcinoma (15), superficial spreading melanoma (16, 17), and uveal melanoma (18); however, a mechanistic connection between CDK4/6 and MAPK pathway activity has not been previously explored. We next investigated the MAPK pathway following CDK4i/6i in our ALM system and observe that although acute palbociclib treatment led to reduced activity of downstream CDK4/6 substrates (pRb, FOXM1) and E2F effectors (PLK1, cyclin A), a robust hyperactivation of MAPK signaling (pERK) and increased downstream cyclin D1 expression were observed across our ALM panel (Fig. 2A). Hyperactivation of pERK and increased cyclin D1 expression were also observed following pharmacological inhibition of CDK4/6 with abemaciclib, ribociclib and genetic silencing of CDK4/6 (**Supplemental Fig. 3A, 3B**).

We next tested whether targeting the MAPK pathway with a MEK1/2 inhibitor (MEKi, trametinib) could ablate intrinsic CDK4i/6i resistance. Combination treatment with MEKi and CDK4i/6i decreases cell cycle proteins (FOXM1, p-Rb, cyclin D1, PLK1) and induces apoptosis (cleaved PARP) to a greater extent than what was achievable by either compound as a single-agent (Fig. 2B). Targeting the MAPK pathway at the level of ERK also increased the cell cycle arrest capacity of CDK4i/6i (**Supplemental Fig. 3C**). Combination treatment with MEKi and CDK4i/6i increased the 3D cytotoxicity in ALM spheroids (Fig. 2C) relative to single-agent CDK4i/6i treatment alone. Further, concurrent MEKi + CDK4i/6i conferred the greatest antitumor durability in long-term colony formation assays (Fig. 2D), and most efficiently reduced the subpopulation of EdU^+^ ALM cells relative to single-agent treatment alone (Fig. 2E).

It was previously reported that reactivation of the MAPK pathway following BRAFi in *BRAF*^V600E^ mutant superficial spreading melanomas was driven, in part, by reduced expression of proteins that negatively regulate the pathway, including members of the Sprouty (SPRY) dual specificity phosphatase (DUSP) family (19). In our ALM cell line panel, we observe that CDK4i/6i treatment decreases protein expression of DUSP4 levels, not SPRY2 or DUSP6 (Fig. 2A, **Supplemental Fig. 3D**). We next tested the hypothesis that alterations in DUSP4 protein levels could be contributing to the hyperactivation of the MAPK pathway following CDK4i/6i. Overexpression of DUSP4 ablates the activation of ERK and induction of cyclin D1 expression following CDK4i/6i (Fig. 2F), and increases the antiproliferative efficacy of CDK4i/6i, as evidenced by the greatest reduction of cell cycle machinery and improved inhibition of EdU positivity relative to CDK4i/6i treatment alone (Fig. 2G).

The MAPK pathway has been experimentally shown to regulate cyclin D1 in melanocytes and *BRAF*^V600E^ superficial spreading melanoma (20), however, the connection between MAPK activity and cyclin D1 expression has not yet been established in ALM. Growing ALM cells in nutrient-replete media following serum starvation induces MAPK activity (pERK, pRSK) and downstream cyclin D1 expression, which could be blocked using MEKi or ERKi (VX-11e), demonstrating the MAPK pathway, at least in part, regulates cyclin D1 expression in ALM (**Supplemental Fig. 3E**). We next tested the hypothesis that ERK hyperactivation following CDK4i/6i preserves cellular proliferation by promoting cyclin D1 expression. Genetic silencing of cyclin D1 increased the cell cycle arrest potential of CDK4i/6i, evidenced by further decreased protein expression of pRb, PLK1, and FOXM1 (Fig. 2H). Genetic silencing of cyclin D1 also decreased the EdU^+^ subpopulation relative to what CDK4i/6i alone could achieve (Fig. 2I). In summary, these data demonstrate ALM cells adaptively escape single-agent CDK4i/6i by hyperactivating the MAPK pathway via reduced DUSP4 expression. The hyperactivation of ERK activity maintains the proliferative capacity of ALM cells treated with CDK4i/6i by promoting cyclin D1 expression.

### ERK hyperactivation drives acquired resistance to CDK4i/6i.

**Figure 3:**
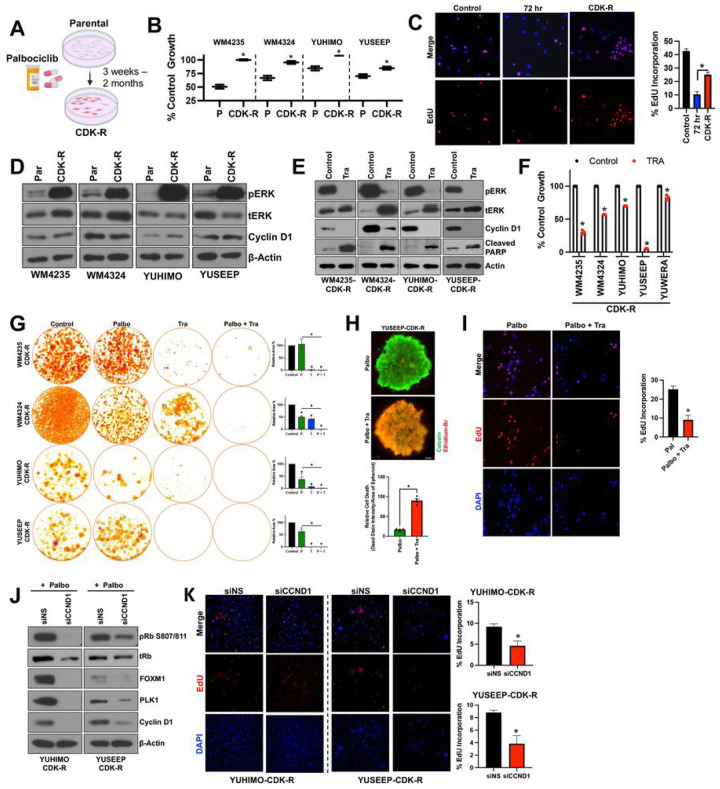
ALMs acquire resistance to CDK4i/6i via ERK hyperactivation. (**A**) ALM cell lines were treated with increasing concentrations of palbociclib (50 nM – 500 nM; up to 2 months) to generate cells with acquired resistance (CDK-R). (**B**) Parental and CDK-R cells were treated with palbociclib (100 nM; 72 hrs) before cell number was quantified by MTT. (**C**) Proliferative capacity was assessed in WM4235 cells treated with palbociclib (500 nM; 72 hrs) and WM4235-CDK-R by EdU staining. Quantitation is shown in the right panel. (**D**) Parental and CDK-R pairs were characterized by Western blotting. (**E**) CDK-R cells were treated with trametinib (10 nM; 24 hrs) while in the constant presence of palbociclib (500 nM) before Western blotting characterization. (**F**) CDK-R cells were treated with trametinib (10 nM; 72 hrs) before cell number was quantified by MTT. (**G**) CDK-R cells were treated with palbociclib (500 nM) and/or trametinib (1 nM) for up to 4 weeks before colonies were fixed and stained with crystal violet. Quantification is shown in the lower panel. (**H**) YUSEEP-CDK-R spheroids were generated, implanted in collagen, and treated with palbociclib +/− trametinib for 72 hours before viability staining and imaging. (**I**) WM4235-CDK-R cells were treated with palbociclib (500 nM) and/or trametinib (10 nM) for 24 hrs before staining with EdU. Quantitation is shown in the bottom left panel. (**J**) Cells were treated with siCCND1 in the presence of palbociclib (500 nM) for protein lysate was immunoblotted. (**K**) Cells were treated as in (**J**) before staining with EdU. *p<0.05 and n^3^3 unless otherwise stated throughout panels.

We next investigated the role of the MAPK pathway in acquired resistance to CDK4i/6i in ALMs. We generated ALM models with acquired CDK4i/6i-resistance (CDK-R) by treating therapy naïve ALM cell lines with increasing concentrations of palbociclib (10–500 nM) between 3 weeks and 2 months (Fig. 3A). CDK-R cells display reduced sensitivity to palbociclib (Fig. 3B) and regain their proliferative (EdU^+^ positivity) capacity (Fig. 3C). Notably, CDK-R cells display elevated phospho-ERK relative to parental cells (Fig. 3D), which functionally drives CDK4i/6i resistance as evidenced by induction of PARP-1 cleavage, reduction in cell cycle proteins, and decreased viability following combination treatment with MEKi (Fig. 3E, 3F). Further, treatment with MEKi resensitized CDK-R cells to long-term treatment with CDK4i/6i (Fig. 3G, **Supplemental Fig. 4A**), induced cytotoxicity in 3D CDK-R spheroids (Fig. 3H) and depleted the proliferative EdU^+^ subpopulation of CDK-R cells (Fig. 3I).

Activation of ERK in the context of acquired CDK4i/6i-resistance has not been reported in melanoma, however evidence in the breast cancer literature proposes a role for *de novo HER2* mutations in estrogen receptor positive (ER^+^) breast cancers (21). We sequenced for *HER2* mutations and copy number variations (CNVs) in our CDK-R models with acquired CDK4i/6i resistance versus their respective therapy naïve parental, and observe no new *HER2* mutations or copy number gains (**Supplemental Fig. 4B**). In addition, evidence in hormone receptor positive (HR^+^) breast cancers suggest potential roles for upstream receptor overexpression (FGFR2) and *de novo* mutations in RAS, AKT1, AURKA, CCNE2, and/or ERBB2 in the activation of ERK following acquired CDK4i/6i resistance (22). We sequenced for *AKT1*, *FGFR2*, *FGFR3*, *FGFR4*, and *NRAS* mutations and CNVs in our CDK-R models versus their respective therapy naïve parental counterparts, and observe no new mutations in these genes. We observed a copy number gain of *NRAS* in the WM4324 CDK-R cells versus its respective parental. No other new copy number gains were observed (**Supplemental Fig. 4B**).

Elevated cyclin D expression was not observed in CDK-R cells relative to their respective parental cell lines. However, in concordance with the role of cyclin D1 in driving intrinsic CDK4i/6i-resistance, genetic silencing of cyclin D1 also resensitized CDK-R cells to palbociclib as evidenced by reduced expression of pRb, FOXM1, and PLK1 (Fig. 3J) and depletion of EdU + cells (Fig. 3K). Altogether, these results indicate that hyperactivation of the MAPK pathway drives acquired CDK4/6 inhibitor resistance, in part, through sustained cyclin D1 expression.

### MEKi increases the in vivo efficacy of CDK4i/6i in therapy naïve and acquired CDK4i/6i-resistant ALM PDX.

**Figure 4:**
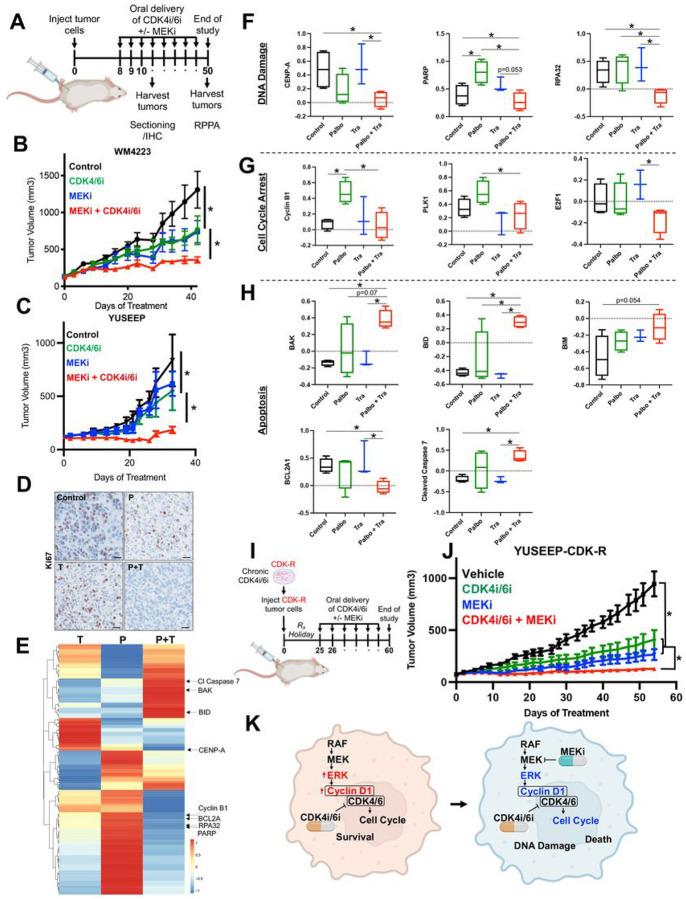
MEKi increases the *in vivo* efficacy of CDK4i/6i in therapy naïve and acquired CDK4i/6i-resistant ALM models. (**A**) Schematic detailing the therapy naïve trial strategy. (**B**) Tumor growth curves of NSG mice implanted with the ALM PDX WM4223 and treated with vehicle control, palbociclib, trametinib, or the combination of palbociclib plus trametinib via oral gavage. (**C**) Tumor growth curves of NSG mice implanted with YUSEEP cells and treated with vehicle control, palbociclib, trametinib, or the combination of palbociclib plus trametinib via oral gavage. (**D**) IHC staining for Ki67 in WM4223 PDX tumor tissue from mice treated for 3 days with vehicle control, palbociclib, trametinib, or the combination of palbociclib + trametinib. (**E**) Heatmap of significant differentially expressed proteins between the palbociclib and control arm, the trametinib and control arm, and the combination palbociclib plus trametinib and control arm. (**F**) Plots depicting differentially expressed DNA repair and damage proteins from the data in (**E**), (**G**) Plots depicting differentially expressed cell cycle proteins from the data in (**E**), (**H**) Plots depicting differentially expressed apoptosis proteins from the data in (**E**). (**I**) Schematic detailing the acquired CDK4i/6i-resistant trial strategy. (**J**) Tumor growth curves of NSG mice implanted with the YUSEEP-CDK-R cells and treated with palbociclib, trametinib, or the combination of palbociclib plus trametinib via oral gavage. (**K**) Graphic summary of the manuscript findings. *p<0.05 and n^3^3 unless otherwise stated throughout panels.

To assess the utility of targeting MEK to increase the *in vivo* efficacy of CDK4i/6i, we implanted the WM4223 patient-derived xenograft (PDX) model, derived from a biopsy of a metastatic ALM that originated in the left foot of a 73 year old male patient, into NOD.Cg-*Prkdc*^*scid*^*Il2rg*^*tm1Wjl*^/SzJ (NSG) mice (Fig. 4A). After 1–2 weeks, tumors were palpable and mice were treated via oral gavage with vehicle control, palbociclib (25 mg/kg), trametinib (0.3 mg/kg) or the combination of palbociclib plus trametinib. Palbociclib and trametinib each conferred significant anti-tumor activity as single-agent treatments; however, the greatest therapeutic benefit was observed in mice receiving combination palbociclib plus trametinib treatment (Fig. 4B, **Supplemental Fig. 5A**). The ALM cell line YUSEEP, derived from the left heel, was also implanted in NSG mice and treated with vehicle control, palbociclib, trametinib, of the combination of palbociclib plus trametinib once tumors were palpable. Combination palbociclib plus trametinib treatment again conferred significantly greater antitumor activity relative to what could be accomplished by the single-agents alone (Fig. 4C, **Supplemental Fig. 5B**). Concurrent treatment with palbociclib and trametinib resulted in the greatest inhibition of cell cycle machinery (i.e., pRb, FOXM1, PLK1, cyclin D1) in lysate collected from a subset of tumor-bearing mice sacrificed after 3 days of treatment (**Supplemental Fig. 5C**) and conferred the greatest decrease of Ki67 staining in tumor tissue (Fig. 4D). At treatment endpoint for the WM4223 *in vivo* study (day 50), tumor tissue was characterized by reverse-phase protein array (RPPA) to identify the mechanism(s) of action underlying the long-term therapeutic efficacy of MEKi + CDK4i/6i relative to single-agent therapy (Fig. 4E). A total of 46 proteins were significantly differentially expressed between vehicle control tumors and combination palbociclib plus trametinib treated tumors that were not observed in the single-agent treated tumors (**Supplemental Table**). Interestingly, a signature indicative of reduced DNA repair capacity (decreased CENP-A, PARP and RPA32 protein expression) correlated with increased double strand DNA breaks in tumors treated with combination palbociclib plus trametinib (Fig. 4F, **Supplemental Fig. 5D)**. Combination palbociclib plus trametinib treatment also resulted in the greatest cell cycle arrest signature (as seen by decreased cyclin B1, PLK1 and E2F1 protein expression (Fig. 4G)), and most significant induction of apoptosis (increased BAK, BID, BIM, caspase 7 cleavage, and reduced BCL2A1 protein expression) (Fig. 4H).

To assess the utility of targeting MEK to overcome acquired resistance to CDK4i/6i *in vivo*, YUSEEP-CDK-R cells chronically treated with palbociclib *in vitro* were implanted in NSG mice (Fig. 4I). In line with observations of reversible (non-heritable) mechanisms of acquired resistance to palbociclib in cholangiocarcinoma cells (23), YUSEEP-CDK-R cells that expanded *in vivo* during a > 3 week drug holiday again exhibited sensitivity to single-agent palbociclib (Figure J). Although vehicle treated YUSEEP-CDK-R tumors exhibited a greater growth rate relative to palbociclib treated, the vehicle treated YUSEEP-CDK-R tumors grew slower than the vehicle treated parental YUSEEP tumors (Fig. 4C). Treatment with MEKi significantly blunted tumor growth of CDK4i/6i-resistant ALM cells, and the combination of CDK4i/6i + MEKi resulted in the greatest antitumor activity and decrease in cell cycle proteins (pRb, cyclin D, PLK1, FOXM1) relative to what could be achieved by the single-agents alone (Fig. 4J, **Supplemental Fig. 5E, 5F**). These findings indicate that continuous pressure from CDK4i/6i is required to maintain maximal vulnerability to MEKi. Altogether, these results underscore the importance of the MAPK pathway in driving intrinsic and acquired CDK4i/6i resistance in ALM and the translational potential of MEKi to increase the *in vivo* antitumor activity of CDK4i/6i against therapy naïve and CDK4i/6i-resistant ALMs, via increased DNA damage, cell arrest and tumor cell death (Fig. 4K).

## Discussion

ALM represents a distinct disease from other forms of CM and the therapy resistance landscape that limits the curability of patients with advanced ALM is poorly understood. ALM remains the most lethal form of CM and existing therapies effective in other forms of CM (e.g., BRAFi, ICB) are not as active in ALM for reasons that are poorly understood. Genetic alterations in the CDK4-pathway occur in the majority of ALM cases, and there is preclinical evidence that ALM cells with CDK pathway alterations are highly sensitive to CDK4i/6i (7). Unfortunately, these findings have not translated clinically, with single agent palbociclib conferring a mPFS of less than 3 months suggesting mechanisms of resistance blunt efficacy. Enthusiasm for the clinical testing of CDK4i/6i-containing therapy strategies in patients with other forms of melanoma has grown in the past decade stemming from a) observations of CDK4-pathway alterations in > 90% of CM cases, b) evidence of downstream activation of CDK4/6 as a consequence of elevated MAPK pathway signaling, and c) a role for CDK4 in ICB resistance (24). Clinical trials have commenced incorporating CDK4i/6i plus MEKi in *NRAS* mutant CM (25, 26), CDK4i/6i plus BRAFi/MEKi in *BRAF* mutant CM (27), and most recently CDK4i/6i plus immune checkpoint blockade (28). Mechanisms of intrinsic resistance to CDK4i/6i have been reported in other forms of melanoma, but no studies have reported CDK4i/6i resistance mechanisms in ALM models.

Here, we report a seminal investigation into the underlying intrinsic and acquired resistance mechanisms that blunt the therapeutic efficacy of CDK4i/6i in ALM. Our study finds that despite the robust inhibition of CDK4/6 substrates and E2F1 effector proteins by single-agent CDK4i/6i, most tumor cells remain viable following treatment with non-physiologically high concentrations of CDK4i/6i that results in a temporary cytostatic response *in vitro* and *in vivo* followed by reactivation of the cell cycle. A recent study proposed a role for elevated CDK6 expression in the intrinsic resistance of NSCLC and superficial spreading melanomas to CDK4i/6i, however we find the opposite in ALM models whereby CDK6 expression directly trends with CDK4i/6i efficacy. It has been proposed that ALM models with CDK4 pathway alterations (defined as *CDK4* gain, *CCND1* gain, *CDKN2A* loss) exhibit elevated sensitivity to CDK4i/6i (7), however, we could not corroborate a differential sensitivity between ALM models based off the status of the CDK4 pathway. This lack of correlation was also reported during the first phase II clinical trial of palbociclib in ALM patients, whereby the authors concluded “neither the genetic status nor the protein expression level of CDK4, CCND1, or CDKN2A was significantly associated with clinical response to palbociclib” (8). Further, we find a notable discordance between the genetic status of the CDK4 pathway and the protein expression of key nodes (e.g., CDK4, CDK6), suggesting both genetic and proteomic characterization of the CDK4 pathway should be performed when stratifying patients for treatment with CDK4i/6i.

We examined the role of MAPK pathway signaling in the context of intrinsic and acquired CDK4i/6i resistance, in part, due to evidence of possible synergies of combination CDK4i/6i plus MAPK pathway inhibitor treatment in breast cancer and superficial spreading melanomas with either *NRAS* or *BRAF* mutations. Our investigation identified a rapid hyperactivation of the MAPK pathway occurs within three days of CDK4i/6i treatment in the context of therapy naïve ALM, which drives downstream expression of cyclin D1. The MAPK pathway hyperactivation and increase in cyclin D1 expression each functionally drove intrinsic CDK4i/6i resistance, which could be reversed by either incorporating a clinically relevant MEK inhibitor (trametinib), an ERK inhibitor, or genetic silencing of cyclin D1. Of note, evidence of MAPK pathway hyperactivation following acute (0–72 hrs) CDK4i/6i treatment has not been reported previously in ALM or other melanoma subtypes. Although MAPK hyperactivation has been reported in other cancer types including head and neck (29) and luminal A breast (30) following acute CDK4i/6i, the underlying mechanism remains poorly understood. We here demonstrate that CDK4i/6i results in decreased expression of the negative regulator of ERK activity, DUSP4. Loss of DUSP4 is functional in the CDK4i/6i-induced activation of ERK, as DUSP4 overexpression ablates CDK4i/6i-induced ERK activation and increases the anti-proliferative activity of CDK4i/6i.

In the context of acquired resistance, we also observed a robust hyperactivation of the MAPK occurs across our panel of CDK-R cells relative to their respective parental lines. Elevated MAPK activity functionally drove resistance in CDK-R cells and could be reversed with the use a MEKi or ERKi. Notably, evidence in ER^+^ breast cancer suggests *de novo HER2* mutations can hyperactivate ERK activity in the context of acquired CDK4i/6i resistance. We confirmed in our paired parental and CDK-R ALM models the lack of *de novo HER2* mutations following acquired CDK4i/6i resistance. Further, it has been observed by others that overexpression of FGFR2 and/or de novo mutations in *RAS*, *AKT1*, *AURKA*, *CCNE*, and *ERBB2* could possibly contribute to MAPK activation following acquired CDK4i/6i resistance. We sequenced for *AKT1*, *FGFR2*, and *NRAS* mutations and CNVs in our CDK-R models with acquired CDK4i/6i resistance versus their respective therapy naïve parental line, and observe no new mutations in AKT1, FGFR2, or NRAS across our CDK-R and parental cell line pairs. We observed a copy number gain of *NRAS* in the WM4324 CDK-R cell versus its respective parental cell line.

Although there was no evidence of elevated cyclin D1 expression in CDK-R cells, genetic silencing experiments reveal CDK-R cells rely on cyclin D1 proliferation. In summary, our findings define MAPK pathway plasticity as an underlying mechanism of intrinsic and acquired CDK4i/6i therapy resistance in ALM. Further, this body of work makes numerous clinically relevant observations for the treatment of patients with advanced ALM including that a) *CDK4* and *P16INK4A* status does not robustly predict ALM patient survival, b) CDK4 pathway alterations do not predict ALM sensitivity to CDK4i/6i, c) CDK6 protein expression does not predict ALM sensitivity to CDK4i/6i, and d) genetic status (e.g., copy number variation) does not correlate with protein expression of CDK4 pathway nodes. These findings provide the rationale to further investigate combination treatment of CDK4i/6i plus MEKi as first- and second-line therapy in patients with advanced ALM.

## Methods

### Cell Culture and Reagents

Melanoma cell lines YUSEEP, YUHIMO, YUWERA, and YUCRATE were obtained from Ruth Halaban (Yale University) in 2020. Melanoma cell lines WM4235, WM4324, WM9, 1205Lu, WM989, WM983B, WM4380 and WM4258, as well as the PDX model WM4223 were obtained from Meenhard Herlyn (Wistar Institute) in 2020. All patient samples were collected under institutional review board (IRB) approval (31). Cell lines were tested for Mycoplasma biannually and authenticated using short-tandem repeat fingerprinting. All cell lines are cultured in RPMI-1640 (Corning,10–040-CM) supplemented with 5% fetal bovine serum (FBS; Cytiva, SH30109.03) in the presence of 5% CO_2_ at 37°C. Commercially purchased compounds include palbociclib (SelleckChem, S1116), ribociclib (SelleckChem, S7440), abemaciclib (Apex Biotechnology, A1794), trametinib (SelleckChem, S2673), AZD6244 (SelleckChem, S1008) and VX-11e (SelleckChem, S7709).

### siRNA Transfection

Cells were transfected for 24 hours with siNS and siCCND1 (Dharmacon, D-001810-10-50) at a final concentration of 20 nMol/L using Lipofectamine 2000 (Invitrogen, 11668–019) transfection reagent. Cells were harvested after 72 hours of knockdown before characterization by immunoblotting.

### Immunoblotting, Cell Cycle Analysis, EdU Staining, and Fluorescent Microscopy

Protein lysates were immunoblotted as previously described (32) with the following antibodies CDK4, CDK6, total Rb, phospho-Rb Ser807/811, PLK1, Cyclin A, Cyclin B1, Cyclin D1, β-Actin, FOXM1, PTEN, p16, phospho-ERK1/2 Thr202/204, total ERK1/2, and cleaved Parp from Cell Signaling Technology. Densitometric analysis was performed by utilizing the Gels function in ImageJ. Individual gel lanes were identified and manually outlined. The band intensity of each gel lane was then plotted by the ImageJ software and the subsequent peaks created were used to quantify the relative protein quantity. The software automatically calculates this based on the area under each peak. The results were then normalized to the protein quantity of β-Actin and control lanes. Fluorescent microscopy was performed as previously described (32) using the manufacturer’s instructions from the EdU kit (Click-iT^™^ EdU Alexa Fluor^™^ 647 Imaging Kit, C10340. For cell cycle analysis, cells were plated at 1×10^5^ per well in a 6-well plated and treated, as indicated. Floating and adherent cells were pooled, pelleted, washed with cold PBS and fixed with 70% ethanol. Fixed cells were subsequently washed, resuspended in PBS, treated with RNase A solution and stained with propidium iodide (0.5 mg/mL, BioLegend, 421301) Cell cycle analysis was performed on Cytek^™^ NL-3000 and data were analyzed using FlowJo Software.

### Cell Viability MTT Assay and Clonogenic Assay

For MTT assays, cells were at 2,000/well in 96-well plates and treated as indicated for 72 hours before thiazolyl blue tetrazolium bromide was added to growth medium, incubated for 4 hours at 37°C, solubilized and color was quantified on a 96 well plate reader (Synergy H1 microplate reader; BioTek) at the absorbance 570 nm. For clonogenic assays, cells were plated at 2–5×10^3^ per well in a 6-well plate and treated twice a week for up to 4 weeks as indicated before colonies were stained with crystal violet. Plates were imaged and quantified by the Colony Area ImageJ plug-in. Individual wells were cropped by the software and thresholds were created automatically to remove the background. Manual cropping and thresholding was performed when image artifacts compromised the software’s ability to properly identify the background. The Colony Measurer function was then used to quantify the percent area covered by cell colonies in each thresholded well.

### Massively Parallel Sequencing

DNA from cell lines was characterized by massively parallel sequencing using a custom-designed, targeted panel as previously described (31, 33).

### Reverse-Phase Protein Arrays

Proteins were isolated from tumor shears and cell lines, and RPPA analysis was performed as previously described (34). Prior antibody testing confirmed the specificity of each antibody, and direct correlation between RPPA and Western blotting results (data not shown). A logarithmic value was generated, reflecting the quantitation of the relative amount of each protein in each sample. Differences in relative protein loading were determined by the median protein expression for each sample across all measured proteins using data that had been normalized to the median value of each protein. The raw data were then divided by the relative-loading factor to determine load-corrected values. Logarithmic values for each protein were mean-centered to facilitate concurrent comparisons of different proteins (34).

### In Vivo Experiments

Xenograft studies were performed using 6–8 week old NSG mice (Charles River Laboratories) in an Association for the Assessment and Accreditation of Laboratory Animal Care-accredited facility. WM4223 (5 × 10^5^) cells or YUSEEP-CDK-R (3 × 10^5^) cells were implanted subcutaneously into NSG mice and stratified into the indicated treatment arms when tumors were palpable (~ 150 mm^3^) to begin treatment. Mice were treated with either palbociclib (25 mg/kg, oral gavage), trametinib (0.3 mg/kg oral gavage) or the combination of palbociclib and trametinib. Tumor sizes were measured every 2 days using digital calipers. Tumor volumes were calculated using the following formula: volume = 0.5 × (length × width^2^).

### TCGA correlation test

In the TCGA (The Cancer Genome Atlas) database, there are 89 skin cutaneous melanoma patients with paired open source RPPA cyclin D1 data and CCND1 mRNA data in primary tumor. Pearson correlation test was performed between cyclin D1 protein level and CCND1 mRNA TPM (transcript per million) level across 89 samples. The null hypothesis of the test is that there is no correlation between the two variables. P value larger than 0.05 indicates we cannot reject null hypothesis. There are 90 skin cutaneous melanoma patients with paired RPPA cyclin D1 data and copy number variation data of the primary tumor in the TCGA. Similarly, person correlation test was performed between cyclin D1 protein level and CCND1 copy number variation across these 90 patients.

### Statistical Analysis

Data show the mean of at least 3 independent experiments. GraphPad Prism 9 statistical software was used to perform Student’s t test where * indicates p < 0.05. Linear mixed models were used to estimate and compare tumor growth rates (mm^3^/day) between treatment groups.

## Data Availability

Copy number variation data of relevant genes for baseline Acral and non-Acral cell lines in Figure 1A, WM4223 tumor median centered protein RPPA data in Figure 4F, G, and H, copy number variation and mutation data of relevant genes for supplementary Figure 4B are stored in supplementary data. Raw RNA sequencing data of baseline Acral melanoma cell lines YUCRATE, YUSEEP, YUHIMO, YUWERA, WM4324, WM4235 generated from this study have been deposited in Sequence Read Archive (SRA) under accession PRJNA953970.

## Statement Of Significance

MAPK activity drives intrinsic and acquired CDK4i/6i resistance. Concurrent targeting of MEK and CDK4/6 reinforces treatment durability and induces a cell cycle arrest and apoptotic program. CDK4i/6i efficacy poorly correlates with expression of ALM driver genes (e.g., CRKL, LZTR1), urging additional investigation of the ALM therapy resistance landscape.
